# Stressors Disclosed on Reddit by Caregivers of Older Adults and Social Support Received: Content Analysis

**DOI:** 10.2196/71452

**Published:** 2025-09-05

**Authors:** Nova Mengxia Huang, Bryan Timothy, Shirley S Ho

**Affiliations:** 1 Nanyang Technological University Wee Kim Wee School of Communication and Information Singapore Singapore; 2 National University of Singapore Yong Loo Lin School of Medicine Centre for Behavioural and Implementation Science Interventions Singapore Singapore

**Keywords:** informal caregivers, Reddit, stressors, social support, optimal matching theory, content analysis

## Abstract

**Background:**

Informal caregiving of an older adult can be a stressful experience. Anonymous online communities, such as Reddit, provide caregivers with an avenue to disclose their stressors and seek support. However, how caregivers disclose their stressors and the effectiveness of these disclosures in eliciting desired social support remain unclear.

**Objective:**

Guided by the stress process model and optimal matching theory, this study examined stressors disclosed by informal caregivers on Reddit and the types of social support they received in response.

**Methods:**

We conducted a quantitative content analysis of posts and comments in 3 subreddits for informal caregivers of older adults. We identified specific stressors disclosed in the posts, analyzed their co-occurrence, and examined the relationship between these stressors and the presence of different types of social support in corresponding comments.

**Results:**

We collected 737 posts and 3446 comments from 3 subreddits. In the posts, caregivers frequently disclosed care recipients’ functional problems (500/737, 67.8%), caregiving relationship strain (279/737, 37.9%), care recipients’ emotional problems (195/737, 26.4%), and a scarcity of health and social resources (98/737, 13.3%). Care recipients’ functional problems often co-occurred with caregiving relationship strain (*χ*^2^_1_=37.2; *P*<.001) and with care recipients’ emotional problems (*χ*^2^_1_=49.0; *P*<.001). Care recipients’ emotional problems also often co-occurred with caregiving relationship strain (*χ*^2^_1_=87.2; *P*<.001). Caregiving activity issues (odds ratio [OR] 1.75, 95% CI 1.06-2.89; *P*=.03) and social role conflict (OR 1.64, 95% CI 1.14-2.36; *P*=.008) were positively associated with informational support received in comments, while care recipients’ functional problems (OR 1.65, 95% CI 1.32-2.08; *P*<.001) were positively associated with emotional support. Disclosing role overload (OR 1.86, 95% CI 1.03-3.36; *P=*.04) and social role conflict (OR 1.95, 95% CI 1.27-2.77; *P*=.002) increased the chance of receiving esteem support, while disclosing social restriction (OR 2.41, 95% CI 1.03-5.60; *P=*.002) increased the likelihood of receiving network support. In addition, we found that nearly no tangible support (3/3446, 0.09%) was provided in response to self-disclosed stressors.

**Conclusions:**

Caregivers frequently disclosed primary and secondary stressors as well as stressors from the social environment, with various primary stressors usually co-occurring in Reddit posts. These stressors varied in their effectiveness in eliciting different types of social support in comments. Managers and moderators of online communities, such as subreddits, are expected to encourage caregivers’ sharing of stressors and promote the exchange of social support with their peers. Health professionals and social workers should develop targeted support interventions to meet the needs of informal caregivers of older adults.

## Introduction

### Background

Informal caregiving for older adults has become increasingly prevalent due to the growing demand for long-term care in aging societies [1]. Informal caregiving refers to the usually unpaid provision of care to a relative or friend who has a chronic illness, disability, or other long-term health and care needs [2]. Those who assume the role of informal caregivers are most often the person’s family members, friends, or neighbors [2,3]. In 2022, a total of 24.1 million American adults served as informal caregivers for family members aged ≥65 years [4]. While informal caregiving is a common experience that may help people gain enrichment and growth in the caregiver role [5,6], individuals face multiple stressors in the process of caregiving. These stressors are aversive life events or conditions that can lead to negative emotions and psychological distress [7]; for instance, informal caregivers of older adults often report financial burdens; work disruptions; and insufficient support from their existing social networks, such as family and friends [8,9]. If not effectively addressed, these stressors can heighten informal caregivers’ stress, negatively impacting their well-being [8-10].

The rise of online communities across social media platforms has provided informal caregivers with alternative avenues for coping with stressors [11,12]. Unlike offline support networks, including service providers, families, and friends, online communities offer informal caregivers access to a broader network of individuals with similar experiences, often with greater ease of access [13,14]. Among these, Reddit stands out for its unique features and popularity among informal caregivers. As an assemblage of community forums, Reddit enables users to engage in discussions on a specific topic within peer-to-peer communities (ie, subreddits) [15]. The anonymous nature of Reddit encourages users to discuss sensitive topics freely without fear of judgment, making it easier for caregivers to disclose their challenges [16,17]. Research has shown that Reddit is widely used by informal caregivers to discuss their problems and seek support from peers [18,19]. Therefore, discussions on Reddit provide a novel and valuable source of data for understanding the stressors informal caregivers face and their support needs.

Although a body of empirical studies has investigated informal caregivers’ communication on Reddit, such as the topics discussed among informal caregivers for people with Alzheimer disease and related dementias [18,19], research on how informal caregivers of general older adults disclose stressors remains limited. According to the stress process model (SPM), caregivers face distinct stressors, including primary and secondary stressors, across different caregiving contexts [20]. Primary stressors refer to sources of stress directly related to acts of caring for care recipients [20], including objective aspects such as managing care recipients’ diseases and symptoms and subjective aspects such as caregivers’ feelings of overload or the loss of value [21]. By contrast, secondary stressors are those indirectly related to caregiving tasks but may lead to caregivers’ stressful experiences; for example, caregivers often encounter conflict with other family members, financial burdens, and social restrictions due to caregiving [20,22]. Later research also identified additional stressors from the social environment, such as a lack of support and resources from their existing social networks [23]. Given that these various stressors may overwhelm informal caregivers of older adults physically and mentally [20], and there is still limited understanding of specific stressors faced by these informal caregivers, it is critical to examine how this caregiver population discloses these stressors in online communities such as Reddit.

Moreover, the SPM posits that caregiver stressors have a complex and interconnected nature, where one chronic stressor can lead to additional stressors over time as caregivers provide long-term care [20]. Several studies have provided empirical support for this premise; for instance, Koumoutzis et al [24] found that informal caregivers’ financial strain was positively associated with their caregiver burden. Similarly, a recent study identified correlations among different challenges disclosed online by informal caregivers of older adults [25]. Despite these findings, further research is needed to deepen our understanding of the relationships among different types of caregiver stressors and to advance the theoretical development of the SPM. In particular, examining how primary, secondary, and social environment stressors interact is essential. A clearer understanding of the co-occurrence and interplay of these stressors can offer a more comprehensive view of the challenges faced by informal caregivers and inform the design of targeted interventions to meet their specific needs.

The SPM also highlights the role of resources, including social (eg, social support) and psychological (eg, self-esteem) resources, in preventing or mitigating the negative impacts of stressors on caregivers’ well-being [20]. While past studies have explored the importance of social support in caregiving contexts [26,27], no studies to date have examined how stressors disclosed online by informal caregivers elicit responses from peers. Studies have found that self-disclosed stressors can reveal an individual’s difficulties and needs, thus serving as a vehicle for obtaining social support [28]: “the assistance that people provide to others when helping them cope with life changes and situational demands” [29,30]. Cutrona and Russell [31] further developed the optimal matching theory (OMT) to delineate different types of social support, namely action-facilitating support and nurturant support. Action-facilitating support, such as informational (advice and informational input) and tangible (providing goods and services) support, could help remove the stressors people experience, while nurturant support, including emotional (empathy and sympathy), esteem (positive expressions of the self and intrinsic values), and network (enhancing sense of belonging) support, could empower people to cope with the stress experienced [26,27].

The OMT also posits that the acquisition of social support depends on the nature of the stressor disclosed during interpersonal communication. The controllability of disclosed stressful events—the extent to which the stress can be prevented or eliminated—predicts the form of social support provided in intimate relationships [31,32]. Highly controllable stressors are more likely to receive action-facilitating support aimed at eliminating the source of stress. However, recent studies have challenged the applicability of the OMT in predicting the types of social support exchanged in online contexts; for example, Yip [33] found that online community users often provided immediate support without carefully considering the perceived controllability of personal stressors. As a result, there might be an incongruence between support seekers’ needs and the types of support provided by respondents. Given the different stressors faced by informal caregivers and the prevalence of their disclosure of stressors in online communities, there is a need to investigate the relationship between the disclosure of specific stressors and the types of support received in response to infer how well the types of social support provided in comments match stressors disclosed in posts [33].

### Objectives

To address these research gaps, this study aims to investigate the interactive dynamics between informal caregivers of older adults on Reddit. This study seeks to examine (1) the different types of stressors disclosed by informal caregivers of older adults in subreddits and their co-occurrence and (2) how the disclosure of various stressors relates to different types of social support received from peers. By conducting a content analysis of caregivers’ posts and other caregivers’ comments on Reddit, we sought to answer the following research questions (RQs):

- RQ1: What are the caregiver stressors most frequently disclosed by informal caregivers of older adults on Reddit?
- RQ2: What caregiver stressors commonly co-occur in posts by informal caregivers of older adults on Reddit?
- RQ3: How do the caregiver stressors disclosed in posts elicit different types of social support (eg, informational, tangible, emotional, esteem, and network support) in the comments on Reddit?

## Methods

### Study Overview

To identify the different stressors disclosed by caregivers and the types of social support provided by other caregivers on Reddit, we conducted a quantitative content analysis. Previous research shows that content analysis helps in understanding the narratives shared by people and uncovering underlying meanings. By analyzing the content of narrative posts in online communities, researchers can gain insights into people’s experiences and emotions related to specific events [30]. Reddit discussion threads, which included the top-level posts and the comments responding to the top-level posts, were used as the unit of analysis.

### Data Collection

We limited our selection to subreddits that discussed the experiences of caring for the general older adult population. While older adults are typically defined as individuals aged ≥60 years, this threshold is not universally fixed in the context of caregiving. Factors such as health status, functional ability, and specific care contexts can influence how “older adult” is defined [34]. Therefore, we did not apply a strict age criterion for older adults in this study.

To identify relevant subreddits, we used the keywords “aging,” “older,” and “elder” combined with “care” and “caregiver.” We then reviewed each subreddit’s description to determine whether it served as a forum for informal caregivers of the general older adult population. While we could not verify the exact identities of caregivers or their care recipients from the posts, subreddit descriptions informed our inclusion criteria. Ultimately, we included 3 subreddits: r/AgingParents (approximately 49,000 members), r/eldercare (approximately 9200 members), and r/elderlycare (approximately 2200 members). Among these, r/AgingParents is one of the largest caregiving-related subreddits, ranking in the top 4% of all subreddits by size. It primarily focuses on the experiences of caring for aging parents. The other 2 subreddits—r/eldercare and r/elderlycare—are designed to support informal caregivers of older adults, including those who may not have a child-parent relationship with their care recipients. Due to the anonymous nature of Reddit, we could not confirm personal details such as the nationalities or ages of the posters. However, the frequent use of US-specific medical terms such as “Medicaid” and “Medicare” suggests that a significant portion of users may be based in the United States.

The posts and their corresponding comments from these 3 subreddits were extracted, and we filtered out the posters’ responses to other comments as well as comments from other users who responded to comments to the posts from the dataset. In addition, posts that were not written by a caregiver and posts that discussed irrelevant content, such as advertisements or news, were excluded from the final sample. This process resulted in a dataset of 1572 posts with 6984 corresponding comments. Due to the large volume of content, we used simple random sampling—a widely accepted method in human-based content analysis [35,36]—to select a representative subset for in-depth analysis. To achieve this, we used a random number generator in Microsoft Excel to select the posts for analysis. We used a 95% CI and a 3% margin of error to determine the sample size required for the study, which was found to be 636 posts. We oversampled by approximately 20% (n=761) to ensure a sufficient number of posts containing self-disclosures of caregiving experiences. Of these 761 posts, 24 (3.2%) that did not include any self-disclosures of caregiving experiences were excluded from the analysis. The final sample analyzed consisted of 737 posts and 3446 comments.

### Coding Scheme

In our content analysis, we examined the stressors from the posts and the types of social support from the comments. The coding scheme for the stressors was based on the SPM and previous works that have examined and identified key stressors that caregivers tend to discuss [20,23]. For the types of social support, we primarily examined the corresponding comments in the dataset. The coding scheme for the different types of social support is based on the social support behavior code in the OMT proposed by Cutrona and Suhr [37], which outlines five different types of social support. Each type was coded as 1 (present) or 0 (absent), as comments and responses can often contain more than one type of social support.

In addition, based on prior studies identifying key factors that influence the receipt of social support [38-40], we also coded for 2 additional variables: support-seeking strategy and the emotional valence of the post. The coding scheme for support-seeking strategy was adapted from Liu et al [39] and included 2 categories: direct requests for support (explicitly asking for help or advice) and indirect requests for support (sharing personal experiences without explicitly asking for help). The emotional valence of the post was categorized into 4 types—positive, negative, neutral, and mixed—based on the sentiments expressed in the posts, as adapted from Li et al [40]. To ensure the fit and suitability of these codes to the dataset, we conducted a preliminary examination with 100 posts and comments. More detailed explanations of the coding scheme, including the definitions and examples of codes, are presented in Tables S1 and S2 in Multimedia Appendix 1.

### Coding Procedures

Two coders independently coded a separate set of 150 posts (representing approximately 10% of the total posts and not included in the final analysis) and 684 corresponding comments. Before beginning intercoder reliability coding, the coders met with the authors to discuss the codebook and clarify what each code entailed. After gaining a good understanding of the codes, the coders initiated the reliability coding process. For codes that failed to achieve acceptable intercoder reliability, the coders discussed and came to an agreement on what the code should cover based on its definitions. After discussion and recoding, the coders achieved moderate to high levels of intercoder reliability (κ=0.85, range 0.75-1.00). Intercoder reliability was calculated using the Cohen κ formula in R with the *tidy-comm* package (version 0.4.1), specifically the test_icr function.

### Data Analysis

To analyze the frequencies of stressors in posts, we conducted a descriptive analysis, including frequency counts and percentages. To investigate the co-occurrence of the identified stressors, we performed chi-square tests for stressors disclosed in posts. We also calculated the number of posts that contained multiple prominent stressors simultaneously to assess their co-occurrence. In addition, we conducted a series of logistic regression analyses to examine whether the presence of a stressor predicted specific types of social support. Support-seeking strategy and the emotional valence of the post were included as control variables in the logistic regression analysis to account for their potential influence on the receipt of social support.

### Ethical Considerations

We obtained approval (IRB-2023-258) from the Nanyang Technological University’s integrity review board before data collection. As this study involved a secondary analysis of publicly accessible data from Reddit and did not involve the recruitment of human participants, it was granted exempt status, meaning that informed consent and participant compensation were not required. To ensure confidentiality and anonymity, all identifiable information was removed from the collected data. These procedures adhered strictly to the guidelines of the university’s integrity review board to maintain ethical standards in research.

## Results

### Caregivers’ Stressors

RQ1 addressed the types of stressors disclosed by informal caregivers of older adults. The results showed that informal caregivers of older adults disclosed a variety of stressors on Reddit, ranging from primary stressors to secondary stressors, as outlined in the SPM. In addition, users disclosed stressors arising from their social environment. Table 1 elaborates on the stressors identified in the Reddit posts, along with their descriptions.

**Table 1:** Caregivers’ stressors, categories, and descriptions, along with post frequencies and percentages (n=737).

Stressors and categories		Description	Posts, n (%)
**Primary stressors**			
	Care recipients’ functional problems	Mention of the care recipient’s health problems or diseases, including their cognitive, behavioral, and physical limitations	500 (67.8)
	Care recipients’ emotional problems	Mention of the care recipient’s mental health issues or negative emotions, such as worry, frustration, and anger	195 (26.4)
	Caregiving activity issues	Mention of issues related to care recipients’ dependence on caregivers regarding caregiving activities, including care recipients’ daily needs (activities of daily living) and complex independent living tasks (instrumental activities of daily living)	76 (10.3)
	Caregiving relationship strain	Mention of a conflict with the care recipient, such as a medical treatment plan	279 (37.9)
	Role overload	Mention of caregivers’ perceived hardships that result from excessive demands anchored in caregiving	22 (3)
**Secondary stressors**			
	Social role conflict	Mention of caregiving demands conflicting with job requirements and strains due to other expectations	108 (14.7)
	Family role conflict	Mention of conflicts among family members, such as unequal division of older adult care and negative relationships between other family members and care recipients	98 (13.3)
	Financial burden	Mention of monetary concerns, including hospital bills, prescription drugs, and physician visit costs	90 (12.2)
	Social restriction	Mention of the impact of the disease or caregiving on caregivers’ social life, such as missing out on social opportunities with friends, family, and others	48 (6.5)
**Stressors from the social environment**			
	Scarcity of health and social resources	Mention of the unavailability of suitable health and social resources, such as limited day care centers or nursing homes	98 (13.3)

The most frequently disclosed stressor was care recipients’ functional problems (500/737, 67.8%), referring to posters’ concerns about care recipients’ health issues or diseases, including their cognitive, behavioral, and physical problems. The second most frequently disclosed stressor was caregiving relationship strain (279/737, 37.9%), referring to conflict between caregivers and care recipients. Caregivers often mentioned problems in communication or feeling misunderstood by the person they took care of. Care recipients’ emotional problems were the third most frequently disclosed stressor (195/737, 26.4%), usually referring to mental health and emotional issues, especially expressions of negative emotions.

The fourth most frequently disclosed stressor was social role conflict (108/737, 14.7%), with caregivers describing how caregiving demands conflicted with their other social roles and led to exhaustion. The scarcity of health and social resources, a stressor from the social environment, was the fifth most prominent stressor disclosed by caregivers, with many posts (98/737, 13.3%) describing the unavailability of suitable health and social resources, such as limited day care centers or nursing homes.

### Co-Occurrence of Stressors

RQ2 examined the stressors that often co-occurred in caregivers’ posts. Table 2 presents the results of the chi-square analysis. To provide additional context and illustrate the dynamics of highly correlated stressors, representative quotes from relevant Reddit posts are presented in Multimedia Appendix 2.

Care recipients’ functional problems often co-occurred with caregiving relationship strain (*χ*^2^_1_=37.2; *P*<.001). Posts that included both care recipients’ functional problems and caregiving relationship strain accounted for 30.1% (222/737) of the posts. Care recipients’ functional problems often co-occurred with their emotional problems (*χ*^2^_1_=49.0; *P*<.001). Posts that discussed care recipients’ functional and emotional problems accounted for 22.9% (169/737) of the posts. Care recipients’ emotional problems often co-occurred with caregiving relationship strain (*χ*^2^_1_=87.2; *P*<.001). Posts that discussed care recipients’ emotional problems and caregiving relationship strain accounted for 17% (125/737) of the posts. In addition, posts that included care recipients’ functional and emotional problems as well as caregiving relationship strain made up 15.3% (113/737) of the posts.

**Table 2:** Chi-square tests for associations between stressors disclosed in caregivers’ posts (n=737).

Variables		Care-recipients’ functional problems	Care-recipients’ emotional problems	Caregiving activity issues	Role overload	Caregiving relationship strain	Social restriction	Social role conflict	Family role conflict	Financial burden	Scarcity of health and social resources
**Care-recipients’ functional problems**											
	Chi-square (*df*)	—^a^	49.0 (1)	0.001 (1)	0.003 (1)	37.2 (1)	5.5 (1)	12.7 (1)	8.2 (1)	0.2 (1)	7.3 (1)
	*P* value	—	<.001	.99	.96	<.001	.02	<.001	.004	.66	.007
**Care-recipients’ emotional problems**											
	Chi-square (*df*)	49.0 (1)	—	0.4 (1)	12.2 (1)	87.2 (1)	0.08 (1)	7.3 (1)	1.1 (1)	0.05 (1)	0.5 (1)
	*P* value	<.001	—	.53	<.001	<.001	.78	.007	.30	.83	.47
**Caregiving activity issues**											
	Chi-square (*df*)	0.001 (1)	0.4 (1)	—	0.8 (1)	1.9 (1)	0.02 (1)	0.4 (1)	2.5 (1)	28.4 (1)	26.6 (1)
	*P* value	.99	.53	—	.37	.17	.89	.55	.11	<.001	<.001
**Role overload**											
	Chi-square (*df*)	0.003 (1)	12.2 (1)	0.8 (1)	—	11.5 (1)	5.1 (1)	5.2 (1)	1.8 (1)	2.3 (1)	3.5 (1)
	*P* value	.96	<.001	.37	—	<.001	.02	.02	.19	.13	.06
**Caregiving relationship strain**											
	Chi-square (*df*)	37.2 (1)	87.2 (1)	1.9 (1)	11.5 (1)	—	15.5 (1)	23.7 (1)	3.8 (1)	4.0 (1)	1.7 (1)
	*P* value	<.001	<.001	.17	<.001	—	<.001	<.001	.05	.047	.20
**Social restriction**											
	Chi-square (*df*)	5.5 (1)	0.08 (1)	0.02 (1)	5.1 (1)	15.5 (1)	—	48.3 (1)	19.7 (1)	19.1 (1)	1.6 (1)
	*P* value	.02	.78	.89	.02	<.001	—	<.001	<.001	<.001	.20
**Social role conflict**											
	Chi-square (*df*)	12.7 (1)	7.3 (1)	0.4 (1)	5.2 (1)	23.7 (1)	48.3 (1)	—	16.8 (1)	24.3 (1)	0.1 (1)
	*P* value	<.001	.007	.55	.02	<.001	<.001	—	<.001	<.001	.75
**Family role conflict**											
	Chi-square (*df*)	8.2 (1)	1.1 (1)	2.5 (1)	1.8 (1)	3.8 (1)	19.7 (1)	16.8 (1)	—	6.5 (1)	4.4 (1)
	*P* value	.004	.30	.11	.19	.05	<.001	<.001	—	.01	.04
**Financial burden**											
	Chi-square (*df*)	0.2 (1)	0.05 (1)	28.4 (1)	2.3 (1)	4.0 (1)	19.1 (1)	24.3 (1)	6.5 (1)	—	4.7 (1)
	*P* value	.66	.83	<.001	.13	.047	<.001	<.001	.01	—	.03
**Scarcity of health and social** **resources**											
	Chi-square (*df*)	7.3 (1)	0.5 (1)	26.6 (1)	3.5 (1)	1.7 (1)	1.6 (1)	0.1 (1)	4.4 (1)	4.7 (1)	—
	*P* value	.007	.47	<.001	.06	.20	.20	.75	.04	.03	—

^a^Not applicable.

### The Relationship Between Stressors and Social Support

RQ3 examined the provision of social support in response to various stressors disclosed in caregivers’ posts. The results of logistic regression showed whether the presence of specific stressors in posts was significantly associated with the presence of different types of social support in the corresponding comments (Table 3).

**Table 3:** Logistic regression of types of social support received on disclosed stressors^a^.

Independent variables: self-disclosed stressors and categories^b^			Informational support				Emotional support					Esteem support					Network support					Tangible support		
			OR^c^ (95% CI)		*P* value		OR (95% CI)		*P* value		OR (95% CI)			*P* value		OR (95% CI)			*P* value		OR (95% CI)			*P* value
**Primary stressors**																								
	Care recipients’ functional problems	1.01 (0.78-1.31)		.94		1.65 (1.32-2.08)		<.001		1.08 (0.78-1.68)			.49		1.02 (0.48-2.11)			.99		1.00 (0.93-1.08)			.98	
	Care recipients’ emotional problems	0.92 (0.70-1.21)		.54		1.19 (0.97-1.46)		.09		1.15 (0.80-1.66)			.46		0.85 (0.40-1.85)			.69		1.00 (0.92-1.09)			>.99	
	Caregiving activity issues	1.75 (1.06-2.89)		.03		0.66 (0.46-0.94)		.02		0.28 (0.09-0.70)			.008		1.02 (0.35-3.09)			.94		1.00 (0.90-1.11)			.99	
	Role overload	0.54 (0.34-0.88)		.01		1.28 (0.86-1.99)		.20		1.86 (1.03-3.36)			.04		1.13 (0.24-5.27)			.88		1.00 (0.83-1.21)			>.99	
	Caregiving relationship strain	0.88 (0.69-1.13)		.33		1.01 (0.83-1.21)		.99		0.92 (0.61-1.20)			.36		.52 (0.27-1.08)			.08		1.00 (0.93-1.08)			.99	
**Secondary stressors**																								
	Social restriction	0.94 (0.64-1.38)		.75		1.15 (0.87-1.53)		.34		0.96 (0.57-1.52)			.77		2.41 (1.03-5.60)			.04		1.00 (0.88-1.13)			.99	
	Social role conflict	1.64 (1.14-2.36)		.008		.88 (0.69-1.12)		.30		1.95 (1.27-2.77)			.002		1.03 (0.45-2.46)			.91		1.00 (0.91-1.10)			.99	
	Family role conflict	0.79 (0.58-1.10)		.16		1.10 (0.87-1.41)		.42		1.32 (0.87-2.01)			.36		0.41 (0.14-1.21)			.11		1.00 (0.91-1.10)			.99	
	Financial burden	1.28 (0.86-1.90)		.22		1.28 (0.99-1.67)		.07		0.91 (0.57-1.53)			.76		1.10 (0.46-2.77)			.80		1.00 (0.90-1.11)			.99	
**Stressors from the social environment**																								
	Scarcity of health and social resources	0.78 (0.55-1.12)		.78		1.24 (0.94-1.63)		.13		1.00 (0.59-1.70)			.99		1.44 (0.59-3.39)			.45		1.00 (0.90-1.11)			.99	

^a^Support-seeking strategy and emotional valence of posts were covariates in the models.

^b^The reference category for each stressor is 0 (absent).

^c^OR: odds ratio.

For action-facilitating support, disclosing caregiving activity issues (odds ratio [OR] 1.75, 95% CI 1.06-2.89; *P*=.03) and social role conflict (OR 1.64, 95% CI 1.14-2.36; *P*=.008) significantly positively predicted informational support. However, disclosing role overload (OR 0.54, 95% CI 0.34-0.88; *P*=.01) reduced the likelihood of eliciting informational support. In addition, we found that nearly no tangible support (3/3446, 0.09%) was provided in response to self-disclosed stressors.

As for nurturant support, disclosing care recipients’ functional problems (OR 1.65, 95% CI 1.32-2.08; *P*<.001) significantly positively predicted emotional support. However, disclosing caregiving activity issues (OR 0.66, 95% CI 0.46-0.94; *P*=.02) had a significant negative effect on emotional support provided in user comments.

In addition, disclosing role overload (OR 1.86, 95% CI 1.03-3.36; *P=*.04) and social role conflict (OR 1.95, 95% CI 1.27-2.77; *P=*.002) increased the chance of receiving esteem support. However, disclosing caregiving activity issues (OR 0.28, 95% CI 0.09-0.70; *P=*.008) had a significant negative effect on esteem support provided in user comments, which was similar to their effectiveness in the elicitation of emotional support. In terms of network support, only disclosing social restriction (OR 2.41, 95% CI 1.03-5.60; *P=*.04) increased the likelihood of the receipt of network support in user comments, with other stressors having no significant association.

## Discussion

### Principal Findings

Using a quantitative content analysis approach, this study examined the stressors disclosed by informal caregivers of older adults on Reddit and the types of social support received in response. Our findings revealed that subreddits were predominantly used by informal caregivers to share primary caregiving stressors, including care recipients’ functional problems, caregiving relationship strain, and care recipients’ emotional problems. These 3 stressors also often co-occurred in caregivers’ posts. Furthermore, the types of stressors disclosed in posts influenced the forms of social support received in the comments. Posts disclosing caregiving activity issues and social role conflicts were positively associated with receiving informational support, whereas posts about care recipients’ functional problems were more likely to elicit emotional support. In addition, we identified positive correlations between social role conflict and esteem support as well as between social restriction and network support.

Notably, care recipients’ functional problems were mentioned in more than half of the posts (500/737, 67.8%), and their emotional issues were discussed in more than one-fourth of the posts (195/737, 26.4%). This finding aligns with previous studies indicating that caregivers of older adults with Alzheimer disease and related dementias often discussed care recipients’ physical and emotional issues on Reddit [18,19]. This implies that care recipients’ physical and emotional well-being is a prominent concern among informal caregivers of older adults across different contexts. In addition, the frequent disclosure of caregiving relationship strain also indicated that caregivers struggled with their relationships with care recipients. Aging-related changes in care recipients’ behaviors make it harder for caregivers to communicate with them, which impacts the quality of the dyadic relationship [41]. Research has found that these primary stressors can significantly predict caregiver burden and impact the quality of caregiving [42-44]. Therefore, interventions are needed to improve caregivers’ health care literacy and communication skills in caregiving.

Social role conflict, a secondary stressor, was also prominent in caregivers’ posts. This finding aligns with prior research showing that social role strain is commonly reported among caregivers of older adults both with and without dementia [23,45]. Caregiving demands for older adults consume substantial time and resources, interfering with other daily activities, such as fulfilling job responsibilities and caring for other family members. Interventions aimed at reducing caregiving demands or helping caregivers balance their various social roles are recommended, as unresolved role conflicts can contribute to caregivers’ mental health issues [46].

Another key finding is the frequent disclosure of a lack of health and social resources, characterized as a stressor from the social environment. Pearlin et al [20] conceptualized access to caregiving resources as a contextual aspect of the stress process. By contrast, this study identified the prevalence of a scarcity of health and social resources as an additional caregiving stressor, directly contributing to caregiver burden. These findings mirror those of Sun [23] and Giebel et al [47], who found that informal caregivers experienced stress from the social welfare system due to a lack of adequate or suitable supportive services, including long-term care facilities. This finding suggests a need to investigate caregivers’ stressors within their social contexts, in addition to primary and secondary stressors.

The co-occurrence of various primary stressors in caregivers’ posts, particularly regarding care recipients’ functional and emotional problems as well as caregiving relationship strain, supports the premise of the SPM that stressors are often interrelated [20]. Previous empirical studies have demonstrated that caregiver stress is a multifaceted process with multiple contributing factors [48]. In the context of caregiving for the general older adult population, caregivers frequently face interrelated challenges such as managing health-related issues, addressing emotional or behavioral changes, and maintaining interpersonal relationships with care recipients [26]. These challenges can result in significant physical and emotional strain, especially as care recipients’ needs become more complex over time [24]. The co-occurrence of multiple stressors underscores the need for further research to explore the specific interactions between caregiver stressors and to develop tailored interventions that address the diverse needs of caregivers for older adults.

When disclosed, caregiver stressors can elicit various forms of social support, such as information, empathy, and encouragement. This aligns with prior research indicating that disclosures of stressful life events on social media platforms are positively associated with receiving social support [49,50]. However, not all stressors showed similar patterns in eliciting social support. According to the OMT, the controllability of disclosed stressful events predicts the type of social support offered [31,32]. Our findings suggest that controllable caregiving-related stressors, such as dealing with logistical issues in caregiving and balancing the caregiver role with other social roles, are more likely to elicit informational support. This might be due to the fact that support providers feel comfortable and knowledgeable enough to provide information based on their personal experiences. However, other controllable stressors, including financial burden and a scarcity of health and social resources, were not significantly associated with informational support. The reason may stem from Reddit’s global community, which limits users’ ability to address stressors that require specific local information or familiarity with local social and health services [51]. Similarly, health-related problems, such as care recipients’ functional and emotional issues, did not effectively elicit informational support. The nonsignificant relationship between health-related problems and informational support may also be attributed to the low perceived control of the support providers [37]. Many caregivers on Reddit might lack confidence in their knowledge of health issues and therefore refrain from offering information. Instead, these users conveyed emotional support in response to the stressors related to care recipients’ health problems.

A notable finding is the imbalance in action-facilitating support provided on Reddit. While informational support was frequently offered in response to caregivers’ disclosures, tangible support was almost entirely absent. This highlights a key limitation of online support communities (OSCs): despite their accessibility and ability to connect diverse groups, they struggle to address caregivers’ practical needs. This aligns with prior research, which also observed limited tangible support in OSCs [39]. One possible explanation is the weak-tie nature of OSCs, which may limit the trust and rapport required for tangible support exchanges [52]. In addition, the global nature of subreddits makes it difficult for caregivers to exchange tangible support because such interactions often require physical proximity [53]. Given these limitations, offline interactions with traditional support networks, such as health care professionals and friends, remain crucial for meeting caregivers’ practical needs.

Investigation into the relationships between caregiver stressors and nurturant support yielded 4 important findings. First, the positive relationship between care recipients’ functional problems and emotional support suggests that users tended to express empathy and concern for caregivers struggling with health care issues. One possible explanation is that emotional support is more commonly provided in response to uncontrollable events, such as the progression of an older adult’s disease [37]. Second, the negative relationships between caregiving activity issues and emotional and esteem support suggest that the self-disclosure of caregiving activity issues is less likely to elicit emotional and esteem support from peers on subreddits. Taking the positive relationship between caregiving activity issues and informational support into consideration, the results align with the framework of the OMT, which posits that highly controllable stressors are more likely to prompt action-facilitating support rather than emotional support. Caregiving activities, such as daily tasks and logistical issues, may be perceived as routine or inevitable aspects of caregiving rather than emotional challenges requiring empathy or validation. As a result, peers in OSCs may prioritize offering practical advice over emotional reassurance.

Third, caregivers’ social role conflicts were more likely to elicit esteem support, which aligns with the needs of support seekers. Previous research has found that conflicts between social roles and the caregiver role are associated with lower self-esteem [54]. The disclosure of social role conflicts in posts may reveal low self-esteem among caregivers, which encourages others to acknowledge their efforts and intrinsic value by providing esteem support. Fourth, caregivers’ stressor related to social restrictions elicited network support from others. The disclosure of social restrictions may reveal caregivers’ need for social networks, prompting users to provide network support by reminding them of the availability of companionship in subreddits. Thus, online communities such as subreddits have the potential to serve as supplementary social networks for caregivers lacking in-person relationships. However, disclosing other interpersonal relationship issues, including family role conflict, was not effective in eliciting network support. This may be due to the complexity of interpersonal relationships in caregiving, especially those involving family dynamics [55,56]. People might feel uncomfortable providing feedback on such issues due to a lack of background information. These findings highlight the importance of considering the context of support transactions when addressing relational stressors.

### Implications

This study has several noteworthy theoretical implications. First, while extant research on informal caregivers’ online communication has primarily focused on either their self-disclosure or social support exchange behaviors, we examined how caregiver stressors disclosed online elicited various forms of social support. This approach enriches the research area by delineating the dynamic of communication in anonymous online communities. Second, this study extends the SPM by providing empirical evidence of stressors from the social environment as well as the co-occurrence of various stressors. The results suggest that future research should aim for a comprehensive understanding of caregivers’ stress process by considering other stressors from the social environment, such as a scarcity of health and social resources, and by examining the complex dynamics among stressors when investigating caregiver stress.

Third, this study challenges the applicability of the OMT in online contexts by providing insights into the extent to which the types of social support received align with individuals’ stressors disclosed on Reddit. The mismatch between disclosed stressors and the social support received, as identified in this research, highlights the importance of recognizing the limitations of online communities in meeting individuals’ support needs. The findings also suggest the need to consider potential boundary conditions when examining the applicability of OMT; for example, future research should consider the context of support communication in addressing caregiver stressors, including the characteristics of the stressors and the online communities where supportive communication takes place.

Practically, the findings of this study can inform social workers and public health practitioners about strategies to meet the needs of informal caregivers of older adults. First, it can inform social workers and public health practitioners regarding the development of targeted interventions to address caregivers’ various stressors. This can include creating caregiving relationship–focused interventions to support high-quality family caregiving, enhancing health and medical education among caregivers, and advocating for more appropriate community-based support resources. Second, managers and moderators of OSCs, such as subreddits, can help create spaces and environments that encourage caregivers’ sharing of stressors and promote the exchange of social support with their peers. Third and last, they could also introduce professionals into online communities to answer caregivers’ questions that peers might not be able to answer, such as those surrounding financial support and health and social resources.

### Limitations

Several limitations of this study should be noted. First, the anonymity of Reddit prevents us from determining crucial contextual information, such as caregivers’ demographics (eg, age and race), country of origin (eg, within or outside the United States), caregiving situation (eg, sole caregivers or sharing caregiving responsibilities with other family members), and the exact age range of care recipients. Without this information, it is difficult to fully understand the specificity and nuances of the struggles experienced by caregivers across these different contexts. Future research should incorporate more contextual information to allow for a better understanding of these unique struggles, which would aid in tailoring supportive interventions for specific caregiver populations. Second, we acknowledge the ongoing debate regarding the use of search terms such as “elder,” which some argue may reflect ageist language [57], although it is commonly used in caregiving contexts. Future research should consider adopting alternative search terms that are inclusive and nonageist. Third, communication patterns on Reddit may differ from those on other platforms, limiting the generalizability of our findings. Future research could compare caregivers’ discussions across platforms to provide a more comprehensive understanding of their stressors. Finally, our content analysis provides descriptive insights but does not address caregiver satisfaction with subreddit discussions or their benefits. Future research should understand users’ perceptions of subreddits and the outcomes associated with subreddit use through interviews, surveys, or other mixed methods.

### Conclusions

This content analysis study found that subreddits for informal caregivers allow users to share their caregiving challenges, including primary and secondary stressors and stressors from the social environment. These disclosures help reveal needs and garner social support from peers. While some controllable caregiving issues effectively elicit informational support, other controllable stressors related to health problems and local policy and service gaps seem to fall short in obtaining the desired informational support. In addition, secondary stressors such as role conflicts and social constraints successfully prompt nurturant support, including esteem and network support, from other users.

## Appendix

Multimedia Appendix 1 Key variables and their descriptions and examples.

Multimedia Appendix 2 Representative quotes with highly correlated stressors.

## Abbreviations

OMT: optimal matching theory
OR: odds ratio
OSC: online support community
RQ: research question
SPM: stress process model

## Data Availability

The datasets generated and analyzed during this study are available from the corresponding author on reasonable request.
