# Supplementary material for: Stressors Disclosed on Reddit by Caregivers of Older Adults and Social Support Received: Content Analysis
Source: JMIR Aging. 2025 Sep 5;8:e71452. doi: 10.2196/71452 (PMC12449668; doi:10.2196/71452)
Supplement: Multimedia Appendix 1 [file aging_v8i1e71452_app1.docx]

**Multimedia Appendix 1**

Tables S1. List of stressors and their descriptions and examples (n=737).

| **Category** | **Description** | Example | **Frequency** | **Percentage** |
| --- | --- | --- | --- | --- |
| ***Primary stressors*** | | | |  |
| Care-recipient’s functional problems | Mention of the care recipient’s health problems or diseases, including their cognitive, behavioral,  and physical limitations | “My 75-year-old mom (10+ year cancer survivor and 2+ year stroke survivor) is wasting away. The radiation treatment she had impacted her ability to produce saliva... I don't know what to do - I feel like I'm watching her gradually die from starvation right before my eyes.” | 500 | 67.8% |
| Care-recipient’s emotional problems | Mention of the care recipient’s mental health issues or negative emotions, such as worry, frustration, and anger | “It was just a terrible shock to her to lose her cat. In retrospect, it seems like her cat's presence was something that she counted on as an anchor. The grief of losing her has just ‘scrambled’ her remaining cognitive abilities to an extent that's alarming” | 195 | 26.4% |
| Caregiving activity issues | Mention of issues related to care recipients’ dependence on caregivers regarding caregiving activities, including care recipients’ daily needs (activities of daily living) and complex independent living  tasks (instrumental activities of daily living) | “I've been taking care of my mom for 10 years, first it started with grocery shopping and some light help, then I became her full-time driver and now I have to cook and clean for her as well as drive her to all of her appointments. She's slowly getting less and less able to care for herself and just driving her to doctor's appointments alone pulls me from work once a week and she never makes them at convenient times for me of course.” | 76 | 10.3% |
| Caregiving relationship strain | Mention of a conflict with the care recipient, such as a medical treatment plan | “Both of them become very upset when I bring this up, and it typically ends in an argument or "I would never dare tell my parents what to do when I was your age." I am feeling very lost, upset, and worried.” | 279 | 37.9% |
| Role overload | Mention of caregivers’ perceived hardships that result from excessive demands anchored in caregiving | “I really feel like I am a prisoner and now my own health is suffering. I am at the end of my rope and my mother will take me down with her.” | 22 | 3.0% |
| ***Secondary stressors*** | | | |  |
| Social role conflict | Mention of caregiving demands conflicting with job requirements and strains due to other expectations | “Going to upend my life to keep an eye on my mom. It may not be forever but it needs to start now. Feeling very guilty and nervous about disappearing from work. Not for the company, but for my coworkers. I know I shouldn't, it's unavoidable and a good reason, but i still do. I also worry about my job, and that makes me feel like a slug.” | 108 | 14.7% |
| Family role conflict | Mention of conflicts among family members, such as unequal division of older adult care and negative relationships between other family members and care recipients | “Some relatives come by with some soup about once a month but I am doing most of the work. I don't mind but when my relatives insinuate that this is my burden to carry alone I start to feel resentful.” | 98 | 13.3% |
| Financial burden | Mention of monetary concerns, including hospital bills, prescription drugs, and physician visit costs | “I am worried about making sure he is as comfortable as possible and not missing out on what life is left. I'll have to figure out how to sell his car and some things not being used and funnel it back into his care needs and or other bills...I have my own debt to deal with as well and cannot get loans or anything due to bad credit.” | 90 | 12.2% |
| Social restriction | Mention of the impact of the disease or caregiving on caregivers’ social life, such as missing out on social opportunities with friends, family, and others | “Just today, I needed to make sure my mother got all her necessary hydration, and I try to make sure she gets it early in the day so she doesn't have to get up to pee in the middle of the night. So I skipped a brunch thing where I would have seen friends.” | 48 | 6.5% |
| ***Stressors from social environment*** | | | |  |
| Scarcity of health and social  resources | Mention of the unavailability of suitable health and social resources, such as limited day care centers or nursing homes | “Many of the facilities we've researched nearby have a minimum age... so we're getting a bit desperate as she continues to progress.” | 98 | 13.3% |

Tables S2. List of social support and their descriptions and examples (n=3446).

| **Category** | **Description** | Example | **Frequency** | **Percentage** |
| --- | --- | --- | --- | --- |
| Informational support | The comments offer information, advice, tips, and suggestions for problem solving | “Have you spoken to her doctor? An appetite stimulant may help...” | 3084 | 89.5% |
| Emotional support | The comments convey empathy, sympathy, encouragement, affection, comfort, and concern | “Hugs. That is a rough situation and you are strong to have stuck it out this long...Take care of yourself ...” | 687 | 19.9% |
| Network support | The comments provide members with access to new companions or remind the person of availability of companions, of others who are similar in interests or experiences | “...I’m afraid I don’t have any advice for you, but wanted you to know that you’re not on your own with this problem...” | 40 | 1.2% |
| Esteem support | The comments express compliment and positive things about the members, and agreement with each other's perspective | “You’re a very thoughtful sibling and I think you’re absolutely right in that they will see your contribution as one less thing to have to worry about...” | 178 | 5.2% |
| Tangible support | The comments offer to give tangible assistance, such as money, materials, or actions | “...now I have a couple things to give away which could be useful for someone in this forum.” | 3 | 0.1% |
