# Supplementary material for: Stressors Disclosed on Reddit by Caregivers of Older Adults and Social Support Received: Content Analysis
Source: JMIR Aging. 2025 Sep 5;8:e71452. doi: 10.2196/71452 (PMC12449668; doi:10.2196/71452)
Supplement: Multimedia Appendix 2 [file aging_v8i1e71452_app2.docx]

**Multimedia Appendix 2**

**Representative quotes with highly correlated stressors**

**Co-occurrence between the care recipient’s functional problems and caregiving relationship strain.**

Example 1: “I moved in to help my 81-year-old mom who has mild memory issues and refuses to do anything for herself anymore. She won’t use a walker, she expects to grab at me and almost make me fall...Anytime I’m in the kitchen or trying to eat, she gabs non-stop about my dad’s illness and death...It’s really bad for my mental health to listen to death stories over and over. She is just senile enough to forget so quickly that I cry anytime she yaps about death and dying. What can I do?”

Example 2: “I came home from university today to take my dad (86) to a GP appointment because the doctor asked me to come just to make sure somebody was actually listening as he refuses to wear his hearing aids..First of all he starts asking why he has this appointment and what the point is. Keep in mind he has swollen, leaking legs and spends 90% of the day complaining about pain and various ailments. Eventually he gets it and I think he'll be ready to go soon. But nope he's in the middle of buying a wheelchair and insists nothing else is more important in that moment. So I have to buy the wheelchair... I'm having a meltdown because we're late, I've just had to stand in a stationary queue for 30 minutes, I'm already and anxious nervous wreck of a person and I'm doing this more or less on my own, my dad decides to ask me "why I'm angry at him because he's unwell". I lose it at this point and my boiling point is reached and I start tearing up and saying how I'm trying to do this all on my own, we're late and I've just had to wait in a 40-minute queue to get his medication. His response to this is essentially "oh well what can we do, it doesn't matter and at least your mother comes round to help me" and that I don't provide him enough company...”

**Co-occurrence between caregiver-recipient’s functional problems and emotional problems:**

Example 1: “How do you get your parents to get their hearing checked? My mother cannot hear anything. She nods and you think she heard you. Then she will ask something I just told her. I tell her I just said that and she says, ‘no you didn’t.’ She denies she has a hearing problem...They get mad when anything is brought up about how they should take care of themselves better or quit denying issues. I’m just not sure what to do at this point. It’s frustrating being around her.”

Example 2: “My parents have been going through a lot for the past few years and it's finally hit a breaking point and I need help. My dad has suffered from Parkinsons for about 5 years now and it's been progressing quickly over the past year with my dad getting dementia, exacerbating everything and making life terribly difficult for both him and my mom. To make matters worse, my brother and I both live far away - we've settled down with families and young (all under 3) kids across the country, making it difficult to get home often to help. My dad's brothers visit occasionally and my high school friends still stop by to check in or help if there is an emergency, but it's still been a LOT for my mom and dad - neither of whom want my dad to go to a mental care or similar facility. We've been asking my mom to talk to her financial advisor and either get paid help at home or look into moving my dad into a mental care facility but they've refused to take those steps (I think in part hoping that my dad's dementia wouldn't progress as fast as it has).Well everything hit a breaking point this week as I mentioned and my mom attempted to commit suicide, taking a lot of pills after a fight with my dad. ”

**Co-occurrence among care-recipient’s emotional problems and caregiving relationship strain.**

Example 1: “For starters, the(y) both have moderate dementia, so there’s that. Dad complains she forgets things and gets angry, meanwhile he does the same thing...whenever my Dad gets super frustrated, he gets mean with me, rings my phone off the hook, pouts, and then will start having delusions. He also was telling her daughter and her husband all sorts BS to get sympathy and they end up buying him stuff and giving him attention. I know people would say, oh the dementia! But this is different. Anyways, that's my little story, lol.”

Example 2: “So my mom is almost 80 and has alzheimer's and every few days she gets yelling mad and frustrated that the island in the kitchen is too big an in the way. She wants someone to cut it down and make it more to her likingI try to explain to her that it works All right and move things so it's less of an annoyance, but eventually she's talking about modifying it again. I'm worried that she'll eventually take matters into her own hands and I do t want that. I also don't want to hunt around for a worker to do it and handle all of that junk or to do it myself. Honestly, she has a bee in her bonnet over nothing. What do I do? I don't see a way to get through to her, she won't listen to reason, but the repeated yelling I difficult. She remembers enough to know that she's wanted to change it for a while and I worry about escalation. Help?”
